# MVAR: A Mouse Variation Registry

**DOI:** 10.1016/j.jmb.2024.168518

**Published:** 2024-03-06

**Authors:** Bahá El Kassaby, Francisco Castellanos, Matthew Gerring, Govindarajan Kunde-Ramamoorthy, Carol J. Bult

**Affiliations:** The Jackson Laboratory, Bar Harbor, ME, USA

**Keywords:** Mouse genome variation, variant annotation, short genetic variants, canonicalization, relational database

## Abstract

The Mouse Variation Registry (MVAR) resource is a scalable registry of mouse single nucleotide variants and small indels and variant annotation. The resource accepts data in standard Variant Call Format (VCF) and assesses the uniqueness of the submitted variants via a canonicalization process. Novel variants are assigned a unique, persistent MVAR identifier; variants that are equivalent to an existing variant in the resource are associated with the existing identifier. Annotations for variant type, molecular consequence, impact, and genomic region in the context of specific transcripts and protein sequences are generated using Ensembl’s Variant Effect Predictor (VEP) and Jannovar. Access to the data and annotations in MVAR are supported via an Application Programming Interface (API) and web application. Researchers can search the resource by gene symbol, genomic region, variant (expressed in Human Genome Variation Society syntax), refSNP identifiers, or MVAR identifiers. Tabular search results can be filtered by variant annotations (variant type, molecular consequence, impact, variant region) and viewed according to variant distribution across mouse strains. The registry currently comprises more than 99 million canonical single nucleotide variants for 581 strains of mice. MVAR is accessible from https://mvar.jax.org.

## Introduction

Model organisms play an important role in investigations of the biological and disease consequences of human genome variation. Bioinformatics resources that support comparisons of mouse and human genotype-to-phenotype data and knowledge are essential to evaluate the functional, phenotype, and disease significance of human genome variation.^1^

Several repositories for human genome variation data and associated annotations for variant consequence and disease relevance are publicly available including ClinVar,^2^ dbSNP,^3^ ClinGen,^4^ the European Variation Archive (EVA),^5^ and gnoMAD.^6^ For the laboratory mouse, the EVA at the European Bioinformatics Institute (EBI) serves as a primary public repository of SNP data for mouse. However, EVA does not include imputed variant calls for mouse, nor does it include expertly curated sequence variants associated with phenotypic alleles. The Mouse Genome Informatics database (MGI) is a comprehensive resource for curated genotype-phenotype associations for the laboratory mouse.^7^ Curated information for variant type and molecular consequence of sequence variants for mouse phenotypic alleles from MGI are available from the Alliance of Genome Resources.^8^ However, molecular consequence annotations for mouse strain polymorphisms are not currently available from MGI. A recently implemented mouse variation resource, GenomeMUSter, aggregates and harmonizes mouse variation from multiple sources and imputes missing genotypes for over 650 different strains at more than 106 million sites.^9^ Although GenomeMUSter is among the most comprehensive mouse genome variation aggregation databases available, the resource does not support a workflow for functional or phenotypic annotation of variants.

The Mouse Variation Registry (MVAR) is a scalable mouse genome variation registry implemented with a microservices architecture to support (i) acquisition of variant from multiple sources, (ii) canonicalization of variants across different sources, (iii) variant annotation, and (iv) data access services through an API and a web application. MVAR was modeled after the ClinGen Allele Registry^10^ which supports the aggregation and exchange of millions of human genome variants for the ClinGen project. MVAR provides unique variant identifiers for mouse genome variants and serves as the source of unique identifiers and variant annotations for the recently released GenomeMUSter resource.^9^ MVAR also integrates sequence variants for genetic alleles with known phenotype consequence and human disease relevance from MGI. Here we describe the design and architecture of MVAR and mechanisms for data access. MVAR is available at https://mvar.jax.org.

## Implementation

### MVAR architecture and data ingest workflow

**Figure 1.**
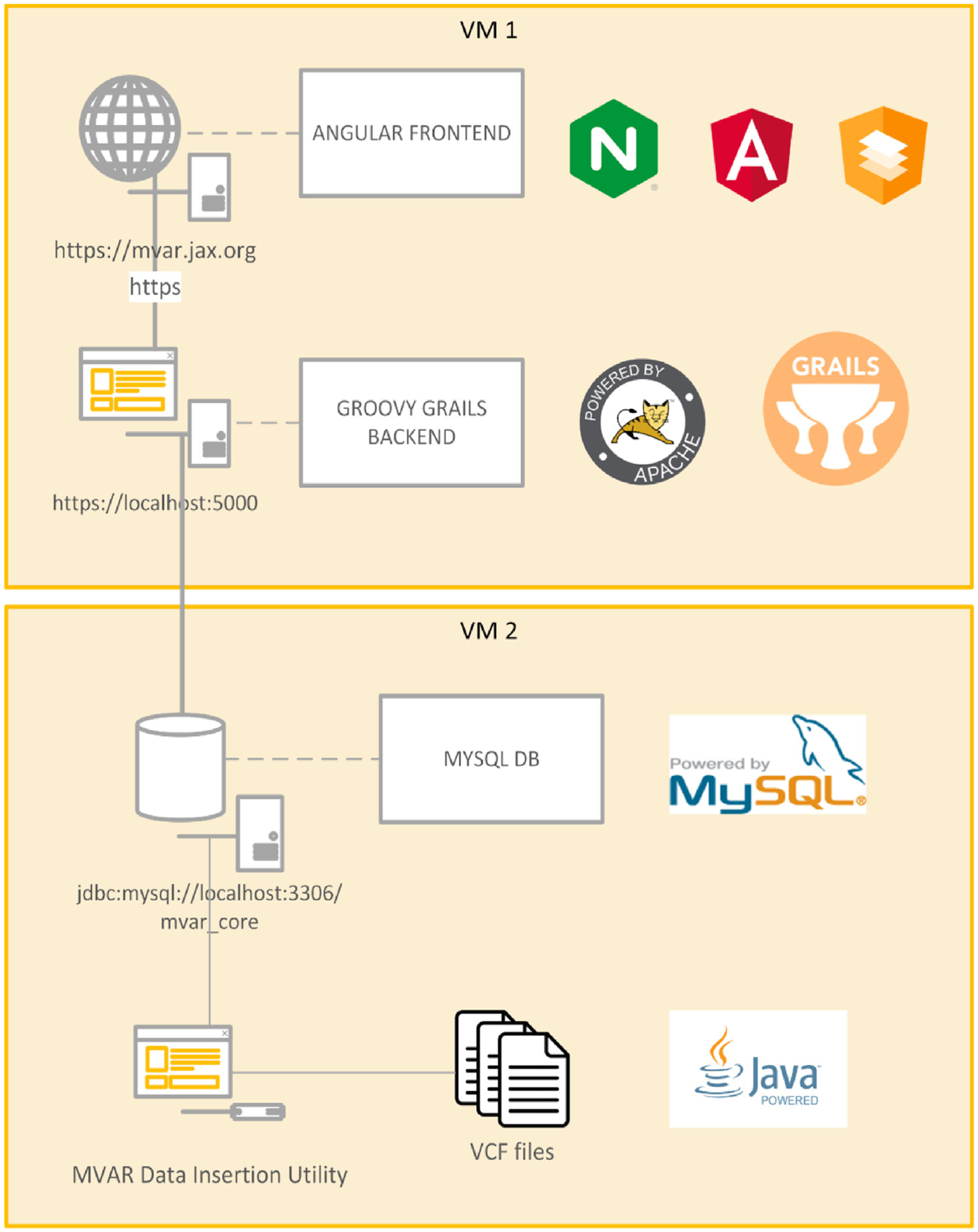
MVAR server architecture and technologies. The frontend is a single-page application built with Node.js and Angular and it uses Angular material for the UI. The Backend is a Grails API (Groovy framework) and enables the data access to the MVAR MySQL database.

A graphical overview of MVAR’s architecture is illustrated in Figure 1 and Supplementary Figure 1. The backend/server application was built with Grails – a Groovy-based web application framework - and the front-end application was built with Angular and the Angular Material UI. Both the source codes are made available on GitHub repositories (https://github.com/TheJacksonLaboratory/mvar-core; https://githb.com/TheJacksonLaboratory/mvar-frontend). The data for MVAR are stored in a MySQL database. The Entity Relation (ER) diagram for the database is shown in Supplementary Figure 2.

The MVAR data ingest workflow (Supplementary Figure 3) normalizes, prepares, and annotates input variation data. The normalization step of the workflow consists of left aligning each variant using the GATK framework developed by the Broad Institute (i.e., shifting the start position of that variant to the left until it is no longer possible to do so).^11^ During that process, the multi-allelic variants (where there is more than one variation in a row) are decomposed so that the results have one variant per row. The next step in the pipeline calls on Ensembl’s Variant Effect Predictor (VEP)^12^ to generate transcript and protein contexts of the variant in Human Genome Variation Society (HGVS) syntax^13^ and links to refSNP identifiers. The final step uses the Jannovar library^14^ to enrich the data with annotations about variant type and molecular consequence. The MVAR database includes a table that stores all of the unique MVAR ids/canonicals and each of these canonical ids points to the corresponding variant in the Variant table. The Variant table has a reference column to store the genome assembly source for a given variant (Supplementary Figure 2).

### Source data

The initial mouse variation dataset used as input into MVAR was for the GRCm38 version of the reference mouse genome assembly downloaded in VCF format^15^ (Sanger REL2005/REL1505) from the Mouse Genomes Project at the Wellcome-Sanger Institute (https://ftp.ebi.ac.uk/pub/databases/mousegenomes/REL-1505-SNPs_Indels/).^16^ This dataset contains about 81 M Single-Nucleotide Variants (SNV), 9 M Deletions and 8 M Insertions (Supplementary Figures 4, 5).

The latest version of MVAR has been updated to genome coordinates from the most recent genome assembly for the C57BL/6J mouse reference (GRCm39).^17^ To update coordinates of variants in MVAR to the new reference genome build, the existing MVAR variant data based on GRCm38 were first mapped to GRCm39 genome coordinates using GATK *LiftOverVCF*. Next the GRCm39 variants were processed using the standard MVAR workflow (Figure 1). The two sets of variants (lifted over from GRCm38 and GRCm39) were compared to identify shared variants and variants unique to each set. The steps in the update processes are outlined below.

### LiftOver of variant coordinates from GRCm38 to GRCm39

To add variants from the new reference mouse genome assembly to MVAR, we first re-processed the GRCm38 data using *LiftOverVCF* to map the variants in MVAR with GRCm38 coordinates to GRCm39 coordinates and re-annotated the re-mapped data set. The four-part workflow is outlined below.

#### a. LeftAlignAndTrimVariant process using the GATK tool (version 4.4)

Although the indels in the GRCm38 variant file were already left aligned, we ran this process to split multi-allelic variants into multiple rows. The reference genome fasta-formatted file was downloaded from the Ensembl archive ftp site (ftp://ftp.ensembl.org/pub/release-102/fasta/mus_musculus/dna/). The sequence dictionary was created using GATK *CreateSequenceDictionary* (Picard) (https://gatk.broadinstitute.org/hc/en-us/articles/13832748622491-CreateSequenceDictionary-Picard). The index was created using Samtools *faidx* (https://www.htslib.org/doc/samtools-faidx.html).


java -jar gatk-package-4.4.0.0-local.jar
  LeftAlignAndTrimVariants --reference Mus_
  musculus.GRCm38.dna_sm.primary_assembly.f
  a --sequence-dictionary Mus_musculus.GRC
  m38.dna_sm.primary_assembly.dict --read-
  index Mus_musculus.GRCm38.dna_sm.primary_
  assembly.fa.fai --variant mgp_REL2005_snp
  s_indels.vcf.gz --output mgp_REL2005_snps_
  indels_aligned.vcf.gz --split-multi-
  allelics


#### b. LiftOver of variant coordinates from GRCm38 to GRCm39

*LiftOverVCF* was used to map the annotated GRCm38 SNPs and indels to GRCm39 genome coordinates. The reference genome (--REFERENCE_SEQUENCE) for GRCm39 was downloaded from Ensembl (ftp://ftp.ensembl.org/pub/release-110/fasta/mus_musculus/dna). The lift over file for GRCm38 to GRCm39 was downloaded from the Ensembl ftp site (https://ftp.ensembl.org/pub/release-110/assembly_chain/mus_musculus/) and the GRCm38_to_GRCm39 chain file was used. Variants that failed the lift over process were written to a separate file.


java -jar gatk-package-4.4.0.0-local.jar
  LiftoverVcf --INPUT mgp_REL2005_snps_in
  dels_aligned.vcf.gz --OUTPUT mgp_REL2005_
  snps_indels_aligned_mm39lifted.vcf.gz --
  CHAIN GRCm38_to_GRCm39.chain --REJECT mgp_
  REL2005_snps_indels_aligned_mm39reject.v
  cf.gz --REFERENCE_SEQUENCE Mus_musculus.G
  RCm39.dna_sm.primary_assembly.fa.gz --
  WRITE_ORIGINAL_ALLELES true --
  WRITE_ORIGINAL_POSITION true


#### c. Annotation with Variant Effect Predictor (VEP)

VEP (version 110, for the GRCm39 release) was used to annotate each variation with the HGVS information at the genomic level. We used genome annotation data from the Ensembl cache (https://ftp.ensembl.org/pub/release-111/variation/indexed_vep_cache/mus_musculus_vep_111_GRCm39.tar.gz). The cache was then unzipped to the default cache folder that VEP uses (~/home/. vep/). VEP was run using the downloaded cache in --offline mode as this process is 9x faster than using the online mode (See VEP documentation for more info).


./vep -i mgp_REL2005_snps_indels_aligned_m
  m39lifted.vcf.gz -o mgp_REL2005_snps_in
  dels_aligned_mm39lifted_hgvs.vcf.gz --
  cache_version 111 --hgvsg --vcf --fasta
  Mus_musculus.GRCm39.dna_sm.primary_assem
  bly.fa --species mus_musculus --
  buffer_size 10000 --assembly GRCm39 --fork
  4 --offline --symbol --merged --
  compress_output bgzip


#### d. Annotation with Jannovar

Jannovar 0.41 was used to annotate the output file above with the transcript databases. The curated transcript database was used (version 110) (https://github.com/charite/jannovar/blob/main/README.md). We used the “download” command in Jannovar to load NCBI’s Refseq transcript database^18^ for GRCm39.


java -jar jannovar-cli-0.41.jar annotate-vcf
  --show-all -d data/mm39_refseq.ser -i mgp_
  REL2005_snps_indels_aligned_mm39lifted_h
  gvs.vcf.gz -o mgp_REL2005_snps_indels_ali
  gned_mm39lifted_hgvs_annotated.vcf.gz


### Processing GRCm39 SNPs and indels

After the coordinates from the GRCm38 variants were lifted over to GRCm39 using steps (a-d) described above, we processed the variant data (SNPs and indels) from the GRCm39 (Sanger REL2021/REL2112) assembly using the standard MVAR workflow. Variant for the build 39 reference assembly were obtained from the EBI website (https://ftp.ebi.ac.uk/pub/databases/mousegenomes/REL-2112-v8-SNPs_Indels/). The separate files for SNPs and indels were evaluated to ensure the headers and contig identifiers were identical using the BCFtools *reheader* program. The two data files were then combined using the GATK *MergeVCF* tool.

Following the processing of GRCm39 variant data with the standard MVAR workflow, we then compared the GRCm39 processing results with the results from the lifted over GRCm38 variant data using BCFTools *isec* (https://www.htslib.org/doc/1.0/bcftools.html#isec). The --output-type parameter was used to produce output as a compressed file using *bgzip*. This process generates reports of intersections, unions, and complements between the two variant datasets. These reports show which variants are in common in the two input data sets and which variants are in one dataset but not the other (Supplementary Figure 6A). The variants exclusive to GRCm39 are inserted as new canonical variants into MVAR; the variants in common are associated with previous MVAR ids. The relationship of the variant to both reference assemblies is also recorded.


bcftools isec -p dir --output-type z mgp_RE
  L2005_snps_indels_aligned_hgvs_annota
  ted_mm39lifted.vcf.gz mgp_REL2021_snps_in
  dels_aligned_hgvs_annotated.rsID.vcf.gz


### Adding new genes and transcripts for GRCm39

Several genes are annotated in the GRCm39 genome assembly that were not present in GRCm38, including *Sts*, *Nlgn4l*, *Akap17a*, and *2510022D24Rik*. These genes are located in the pseudoautosomal region (PAR) shared between the X and Y chromosomes, a region of the assembly that was improved significantly in GRCm39 (see https://ncbiinsights.ncbi.nlm.nih.gov/2020/10/16/refseq-grcm39/). The new genes were added to the MVAR database using MySQL “insert” statements and included the corresponding MGI gene ID, gene symbol, and gene name. New transcripts were added to the variant database during the annotation process using Jannovar and their Ensembl ID is accessible in the “ANN” info ID of a VCF row.

### Insertion of variants into MVAR

The insertion of variants into the MySQL is accomplished using a Java utility program developed to efficiently insert annotated variants as a batch process. The variant files are first inserted through a batch insertion process where a batch number can be set accordingly. Then the variant/strain and variant/transcript relationships are inserted into the corresponding MySQL tables. Supplementary Figure 7 shows a graphical overview the algorithm used for the variant insertion.

The *mvar-utility* program is available from a Github repository (https://github.com/TheJacksonLaboratory/mvar-utility). We modified the program in order to link the new GRCm39 variant to the corresponding existing variant in the GRCm38 reference in MVAR through their canonical ID.

Using MVAR pipeline, we inserted 2851 curated sequence variants associated with phenotypic alleles from MGI available from the Alliance of Genome Resources data download page (https://fms.alliancegenome.org/download/VCF_GRCm39.vcf.gz). The MVAR canonicalization process identified 107 variants out of the 2851 submitted that already exist in mgp_REL2021 (103 SNPs and 4 indels). The 2744 unique new variants from MGI were added to the MVAR database and assigned MVAR identifiers (Supplementary Figure 6B).

### MVAR data access

**Figure 2.**
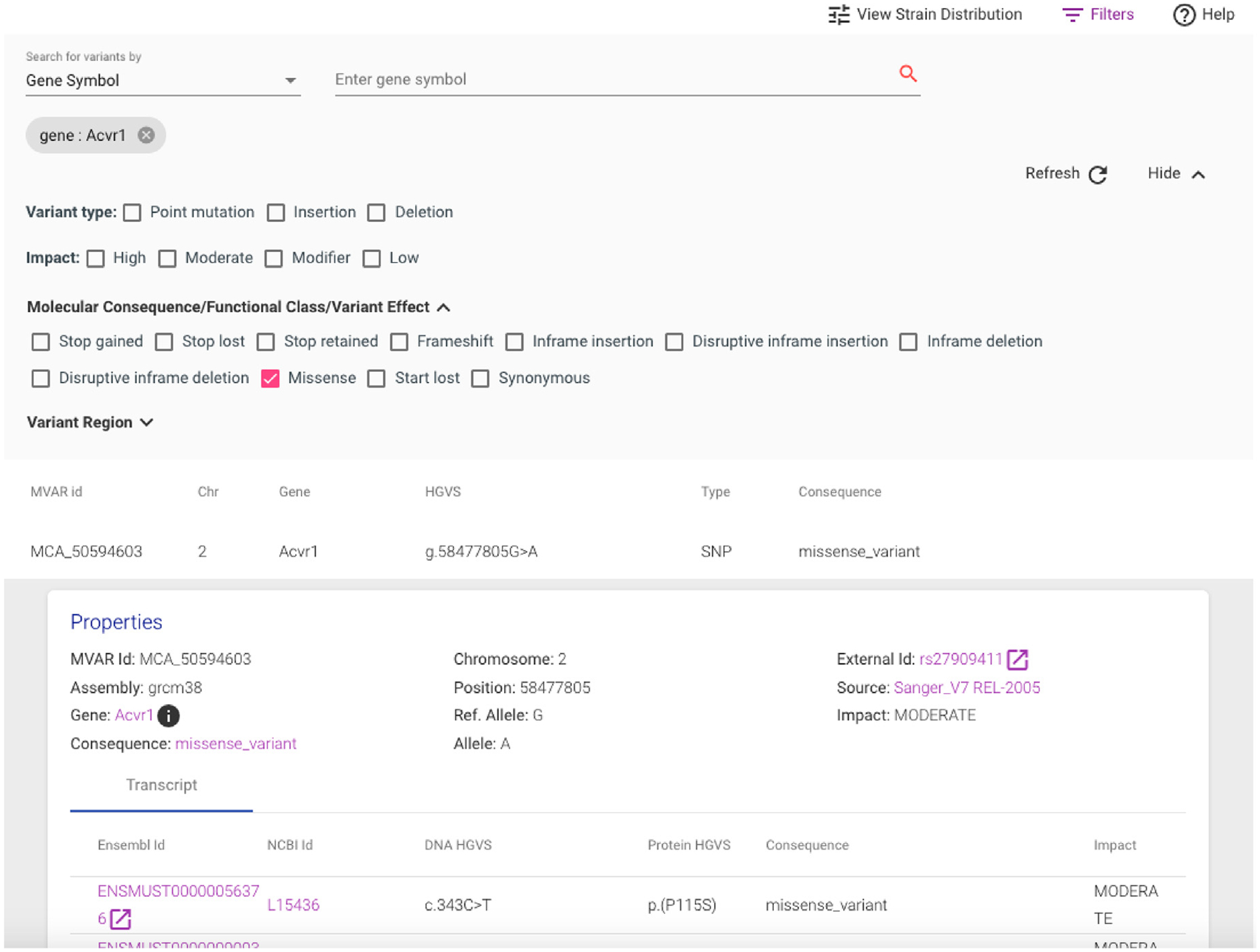
Screenshot of a search result for missense variants in the *Acvr1* gene. The Filters option in the upper right hand corner of the page allows researchers to display only those variants with specific attributes.

**Figure 3.**
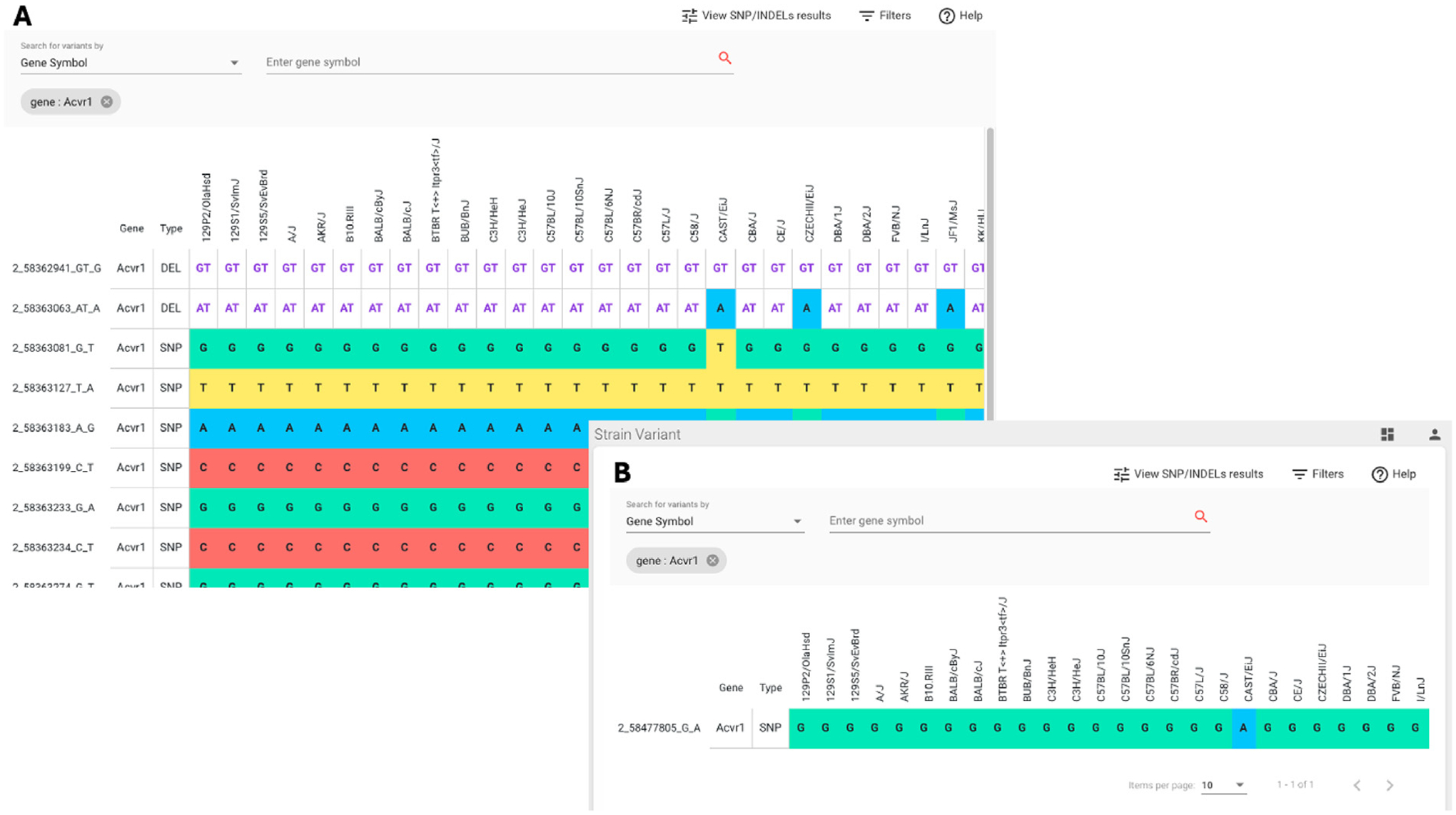
Screenshot of the strain distribution of variants in the *Acvr1* gene. (**A**) Selecting the View Strain Distribution option in the upper right hand corner of the search results page shown in Figure 2 generates a table of variants across mouse strains for the gene of interest. Users can return to the search result summary page by selecting View SNP/INDELs results in the upper right hand corner of the page. (**B**) Variants for the *Acvr1* mouse gene filtered for missense variants using MVAR’s Filters feature.

MVAR supports programmatic data access through an Application Programming Interface (API) for gene, variant, transcript, and mouse strain endpoints (https://mvar.jax.org/mvar/webjars/swagger-ui/3.20.9/index.html?url=/mvar/apiDoc). The API service uses HTTP with JSON data payloads and supports GET, POST, and PUT requests. A web-application implemented with Angular and Grails Groovy supports searches for variants by gene, genome region, variants in HGVS syntax, dbSNP id, and MVAR canonical id (Figure 2). Results are available in both graphical and tabular formats (Figures 2,3). Search results can be filtered by various annotations including: variant type, impact, molecular consequence, variant region, and strain. A graphical display of the distribution of a specific variant across mouse strains can be generated by selecting “View Strain Distribution” (Figure 3).

## Discussion and Future Directions

The MVAR resource was implemented as a series of micro-services, providing both interoperability and ease of software maintenance. Key components of the registry include an annotation service which calculates the type and consequence of mouse genome variants and a service that checks incoming variants to determine if they are equivalent to variants already in the system (i.e., canonicalization). MVAR plays a unique role among mouse genome variation resources in that it can be deployed for annotation of custom variation aggregation database such as GenomeMUSter^9^ that include imputed variant calls and variation data from mouse strains that are not available in all data repositories. MVAR also provides user interfaces that allow researcher to view the strain distribution of variants and to filter the data displayed by variant annotations.

Future plans for MVAR will enhance the resource and add to its unique functionality. A major planned enhancement is the ability to filter variants to display those that have curated associations with specific mouse phenotypes and/or human disease associations from MGI. This functionality will be particularly valuable for evaluating the disease relevance of genes and variants identified in humans. Another planned enhancement is the ability for researchers to upload VCF files to MVAR to identify potentially causative *de novo* mutations in the sequence data for a new strain or experimental mouse model. Finally, future versions of MVAR will incorporate structural variants in the mouse genome (<50 bp)^19^ along with the short variants and indels (>50 bp) that are represented currently.

## Supplementary Material

Supplementary Figures

## Appendix A. Supplementary material

Supplementary data to this article can be found online at https://doi.org/10.1016/j.jmb.2024.168518.
