# Supplementary material for: MVAR: A Mouse Variation Registry
Source: J Mol Biol. Author manuscript; Available in PMC 2026 Mar 24. (PMC13011370; doi:10.1016/j.jmb.2024.168518)

**Supplementary Figures**

**Supplementary Fig. 1.** MVAR Architecture diagram showing the basic workflow, starting with data ingest as VCF formatted files to data access via a Restful API and web application.


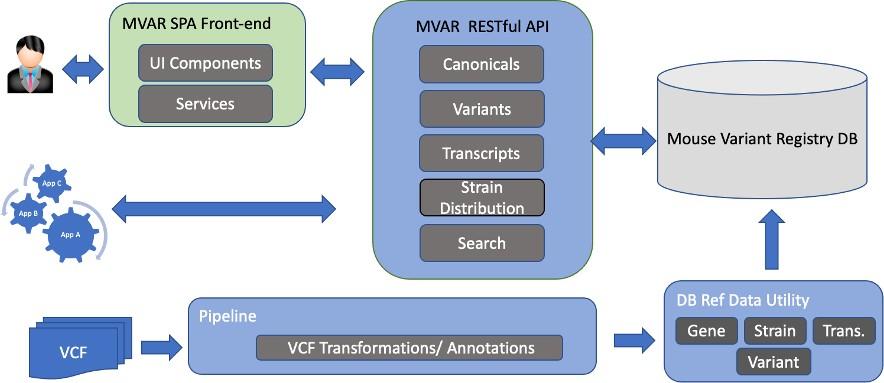


**Supplementary Fig. 2.** Entity Relation diagram of the MVAR MySQL database.


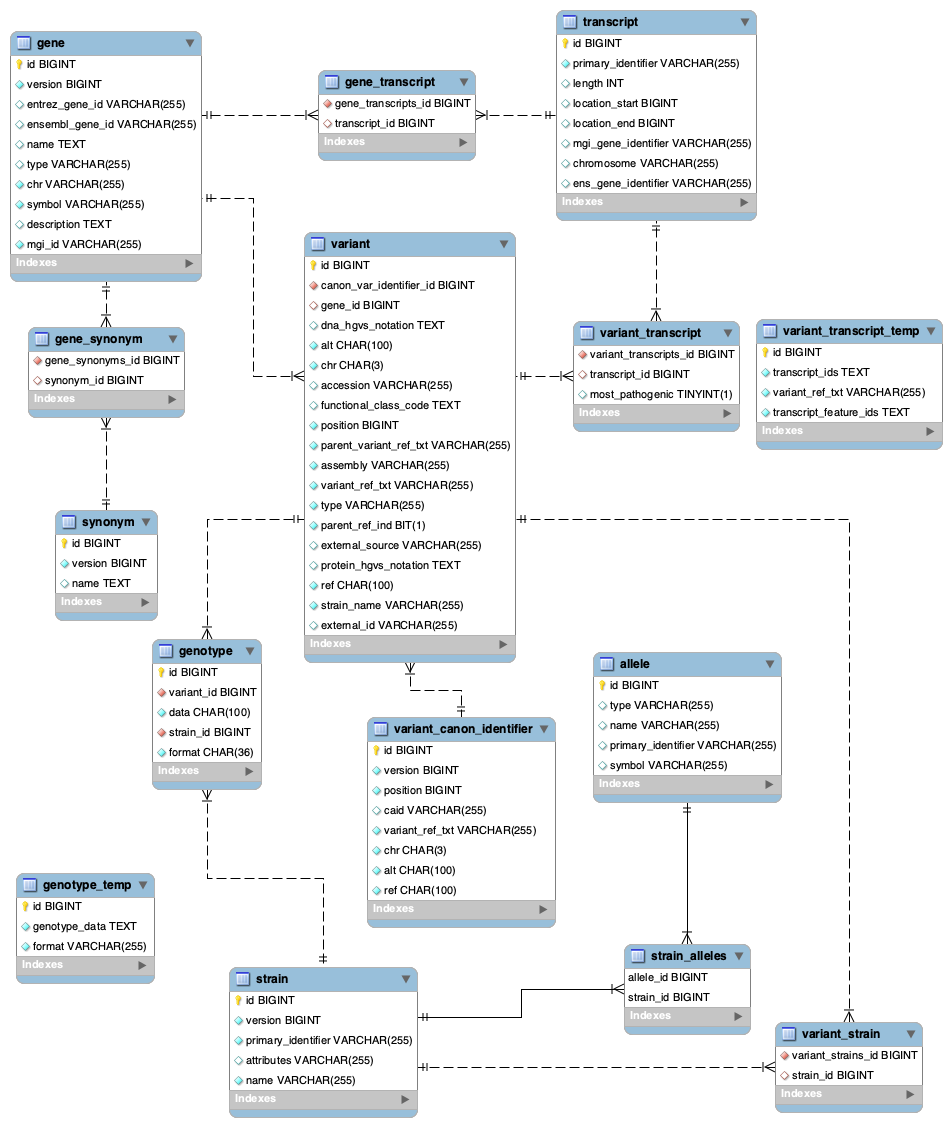


**Supplementary Fig. 3.** Details of the VCF transformations/Annotations component of the MVAR workflow.

#
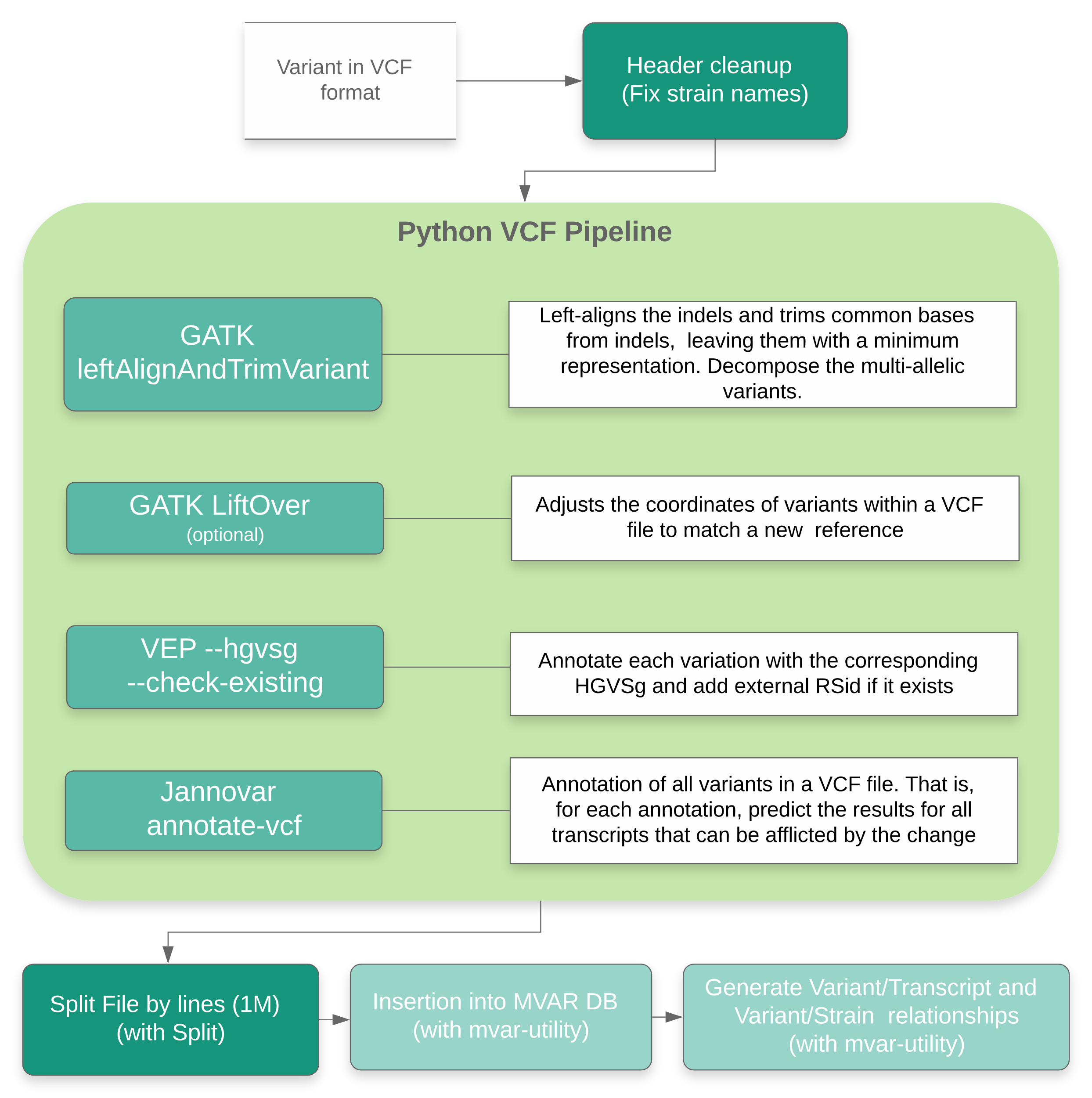


**Supplementary Fig. 4.** Mouse sequence variant distribution across chromosomes for (**A**) Sanger REL2005 and (**B**) Sanger REL2021 after the VEP annotation process.

**
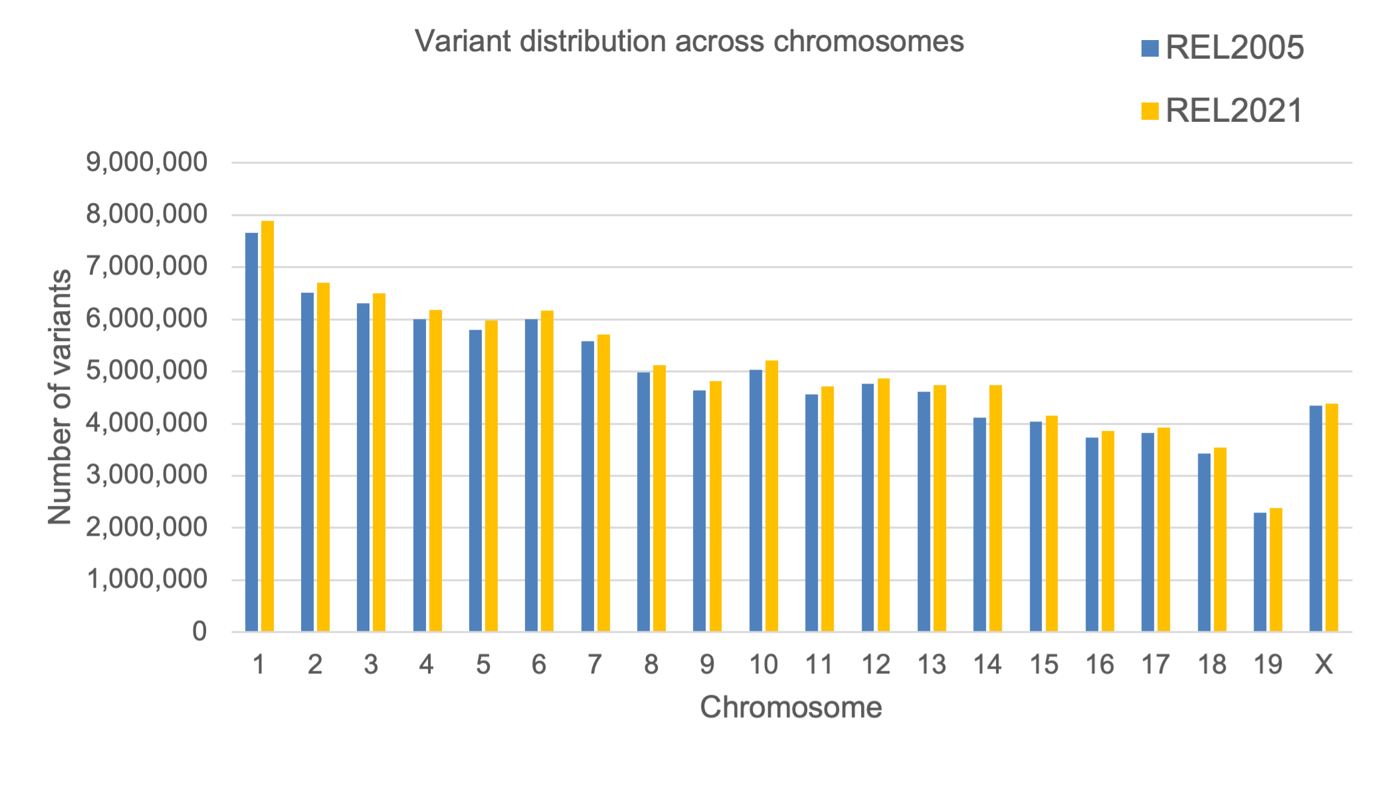
**

| **A.** | **chromosome** | **variants** |  | **B.** | **chromosome** | **variants** |
| --- | --- | --- | --- | --- | --- | --- |
|  | 1 | 7,664,991 |  |  | 1 | 7,884,305 |
|  | 2 | 6,517,915 |  |  | 2 | 6,707,508 |
|  | 3 | 6,310,619 |  |  | 3 | 6,500,569 |
|  | 4 | 6,005,217 |  |  | 4 | 6,179,536 |
|  | 5 | 5,796,516 |  |  | 5 | 5,979,346 |
|  | 6 | 6,007,231 |  |  | 6 | 6,171,352 |
|  | 7 | 5,585,800 |  |  | 7 | 5,708,546 |
|  | 8 | 4,984,090 |  |  | 8 | 5,121,922 |
|  | 9 | 4,639,087 |  |  | 9 | 4,810,169 |
|  | 10 | 5,034,486 |  |  | 10 | 5,210,487 |
|  | 11 | 4,562,897 |  |  | 11 | 4,709,996 |
|  | 12 | 4,760,197 |  |  | 12 | 4,873,218 |
|  | 13 | 4,610,411 |  |  | 13 | 4,738,295 |
|  | 14 | 4,119,099 |  |  | 14 | 4,734,244 |
|  | 15 | 4,032,272 |  |  | 15 | 4,158,938 |
|  | 16 | 3,731,336 |  |  | 16 | 3,855,535 |
|  | 17 | 3,824,927 |  |  | 17 | 3,926,016 |
|  | 18 | 3,429,048 |  |  | 18 | 3,543,167 |
|  | 19 | 2,296,318 |  |  | 19 | 2,385,115 |
|  | X | 4,350,838 |  |  | X | 4,386,318 |

**Supplementary Fig. 5.** Distribution of Variant Classes from (**A)** Sanger REL2005 and (**B**) Sanger REL2021.


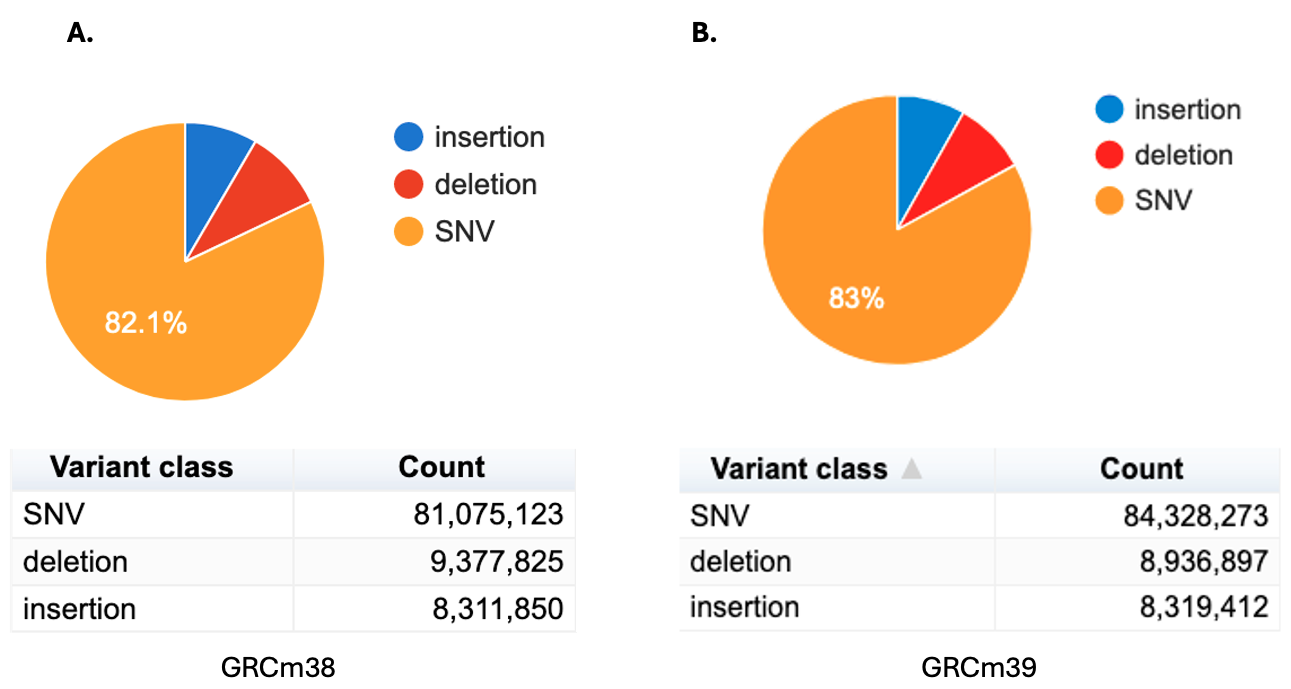


**Supplementary Fig. 6.** (**A)** Histogram of MVAR variant counts generated by BCFtools *isec* showing the variants unique to the REL2005 variant data lifted over from GRCm38 to GRCm39 coordinates, the variants unique to the REL2021 variant data for GRCm39, and the shared variants. (**B)** Distribution of variants between REL2021 and phenotypic allele variants from MGI. The y-axis has a logarithmic scale to better view the plot bars.

**A.**

**B.**

**Supplementary Fig. 7.** Graphical overview of the MVAR variant insertion process. The algorithm is implemented in the *mvar-utility* program in the VariantInsertion class.


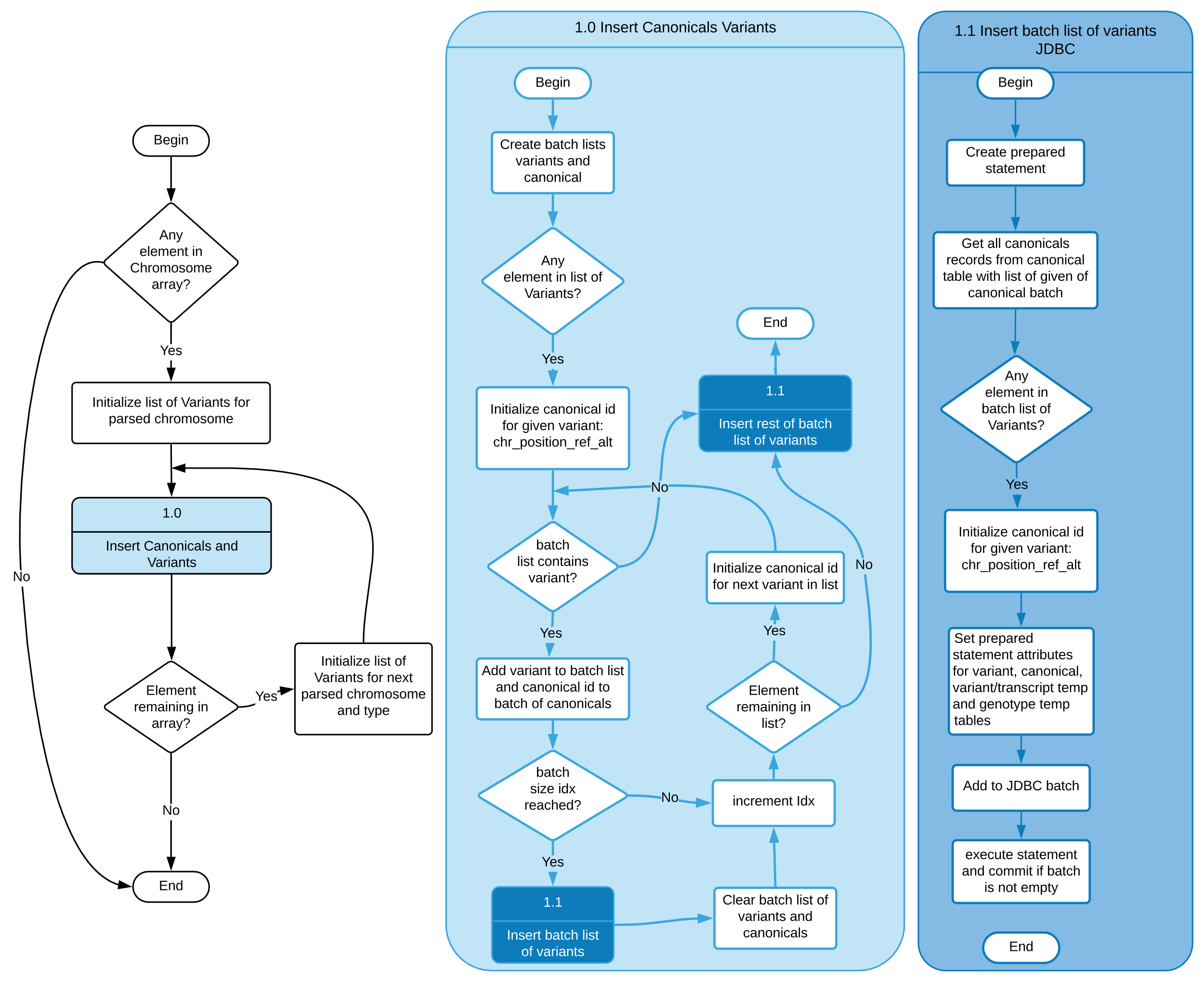

Supplement: Supplementary Figures [file NIHMS2155712-supplement-Supplementary_Figures.docx]
